# Assessment of Egg Yolk Oil Extraction Methods of for ShiZhenKang Oil by Pharmacodynamic Index Evaluation

**DOI:** 10.3390/molecules21010106

**Published:** 2016-01-18

**Authors:** Ping Wu, Yu Pan, Jianye Yan, Dan Huang, Shunxiang Li

**Affiliations:** 1 Hunan Province Engineering Research Center of Bioactive Substance Discovery of Chinese Medicine, School of Pharmacy, Hunan University of Chinese Medicine, Changsha 410208, China; pingwu@xtu.edu.cn (P.W.); panyu1226@gmail.com (Y.P.); yanjianye2015@hotmail.com (J.Y.); huangdan2005@hotmail.com (D.H.); 2 School of Chemistry, Xiangtan University, Xiangtan 411105, China

**Keywords:** egg yolk oil, extraction methods, pharmacodynamic index, ShiZhenKang oil

## Abstract

To assess the extraction methods of egg yolk oil in ShiZhenKang (SZK) oil, which is used to treat eczema, a mice model of eczema was established by using 2,4-dinitrochlorobenzene (DNCB). The therapeutic effects of egg yolk oil extracted by different methods from SZK oil on the model of acute eczema in mice were evaluated. The oil yield rate of ethanol extraction is 42.06%. Its egg yolk oil is orange and has a rich, sweet, egg smell. Moreover, the SZK oil prepared from it has a very good therapeutic effect on the model of acute eczema in mice. The alcohol extraction method is the preferable method according to a comprehensive evaluation of each index of seven kinds of methods to extract the egg yolk oil.

## 1. Introduction

Eczema is a common skin disease in infants, mainly due to overnutrition, dyspepsia, food allergies, external stimulus and genetic factors [1,2,3]. The main clinical findings include intense pruritus, various types of skin injuries and repeated episodes. Full recovery may require prolonged treatment and frequent outpatient visits. It not only represents an economic burden for parents, but is also detrimental to the physical and mental health of infants [4,5,6].

Currently, there are many kinds of treatments for infant eczema, For example, Chinese herbs, which belong to Chinese Traditional Medicine, have been successfully developed to treat infant eczema with relatively few side effects [7]. ShiZhenKang (SZK) oil, another Chinese medicine developed by the Hunan Academy of Chinese Medicine Affiliated Hospital, has shown good curative effects on infant eczema without side effects [8]. Moreover, SZK oil could renew aging skin [9]. However, the main component in SZK oil—the egg yolk—when prepared by traditional methods suffers from many disadvantages, such as a black color, disgusting odor, low bioavailability, easy to wipe off by patients, staining of clothes and unstable nature. Therefore, it is necessary to improve the methods for extracting egg yolk oil [10,11].

At present, there are many extracting methods for egg yolk oil, such as the dry distillation method, baking method, reduced pressure distillation, solvent extraction method, supercritical CO_2_ extraction method, sub-critical propane extraction method and enzymatic methods [12,13,14]. Each of them has their own disadvantages. For example, supercritical CO_2_ extraction methods are expensive and require expensive equipment [15]. The phospholipid content of egg yolk oil extracted by the sub-critical propane extraction method is low, and needs further extraction by a mixed solution of methanol and chloroform, thus it is also used rarely [16,17,18]. Besides, the explosivity of propane is also a potential risk. As for the enzymatic method, the product contains fatty acids [19]. Currently, the most commonly used industrial methods are the dry distillation method and solvent extraction method. However, there is a general lack of research on both methods [20,21]. In this study, the dry distillation method, baking method, reduced pressure distillation, and solvent extraction method were used to obtain egg yolk oil which was then used to prepare SZK oil. The effects of the different methods on egg yolk oil extraction were evaluated and the character and oil yield rate were also used to evaluate the optimal extraction method. Moreover, mice models of acute eczema were established to study the treatment efficiency using SZK oils prepared by the different methods. 

## 2. Results

### 2.1. Color, Smell and Oil Yield Rate of Egg Yolk Oil Extracted by Different Methods

**Figure 1:**
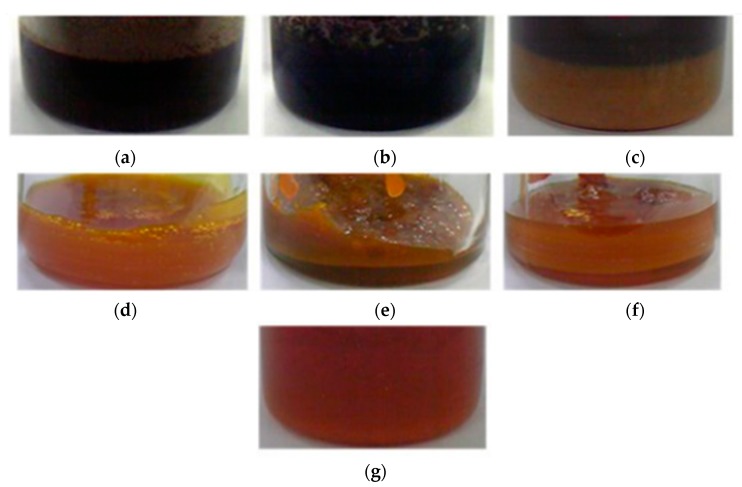
Images of egg yolk oils extracted by seven different methods: (**a**) dry distillation method; (**b**) baking method; (**c**) reduced pressure distillation method; (**d**) ether extraction method; (**e**) ethanol extraction method; (**f**) petroleum ether extraction method and (**g**) chloroform extraction method.

The egg yolk oils extracted by dry distillation, baking, and reduced pressure distillation were brown and disgusting (Figure 1a–c). The oils extracted by the ether, ethanol, petroleum ether, and chloroform extraction methods were orange and egg fragrant (Figure 1d–g). The oil extracted by the ethanol extraction method was very fragrant. The oil yield with the chloroform extraction method was the highest, followed by the petroleum ether method, drying distillation method and ethanol extraction method (Table 1).

**Table 1:** Color, smell and oil yield rate of egg yolk oils extracted by different methods.

Groups	Color	Smell	Oil Yield Rate (%)
Dry distillation method	Black brown	Empyreumatic	34.14 ± 3.51
Baking method	Black brown	Empyreumatic	49.10 ± 2.32
Reduced pressure distillation	Brown	Empyreumatic	23.51 ± 1.34
Ether extraction method	Orange	Slight egg fragrance	35.00 ± 3.62
Ethanol extraction method	Orange	Strong egg fragrance	42.06 ± 5.35
Petroleum ether extraction method	Orange	Slight egg fragrance	56.79 ± 5.68
Chloroform extraction method	Orange	Slight egg fragrance	57.74 ± 4.53

### 2.2. Pharmacodynamic Index of Egg Yolk Oils Extracted by Different Methods

The mouse eczema model was established by application of 2,4-dinitrochlorobenzene (DNCB) solution to the dorsal skin of mice before administration of the different egg yolk oils. According to the visual inspection of the mice’s backs, the mice had varying degrees of eczema, which indicated that the model was established successfully. The eczema symptoms of the reduced pressure distillation group worsened three days after the administration and aggravated obviously five days after the administration, while the eczema symptoms of the other groups began to improve. The curative effect was similar to that obtained with paeonol (control drug group) (Table 2).

**Table 2:** The influence of drugs on the stimulated part skin of mice backs ($\bar{X}\pm \mathrm{SD}$).

Groups	Assessment		
	Pre-Administration	Administration for 3 Days	Administration for 5 Days
Blank control group	0	0	0
Model control group	1.00 ± 0 *	1.00 ± 0 *	1.00 ± 0 *
Dry distillation group	1.25 ± 0.46 *	1.00 ± 0	0.13 ± 0.035 ^Δ^
Baking method	1.37 ± 0.52 *	0.88 ± 0.35	0.38 ± 0.052 ^Δ^
Reduced pressure distillation group	1.25 ± 0.46 *	1.50 ± 0.53 ^Δ^	3.00 ± 0.023 ^Δ^
Ether extraction group	1.50 ± 0.76 *	0.50 ± 0.23 ^Δ^	0.25 ± 0.046 ^Δ^
Ethanol extraction group	1.13 ± 0.64 *	0.56 ± 0.37 ^Δ^	0.26 ± 0.046 ^Δ^
Petroleum ether extraction group	1.00 ± 0.53 *	0.75 ± 0.46	0 ^Δ^
Chloroform extraction group	1.43 ± 0.98 *	0.86 ± 0.38	0.14 ± 0.038 ^Δ^
Paeonol control group	1.12 ± 0.75 *	0.92 ±0.35	0.25 ± 0.046 ^Δ^

Note: each group compared with blank control group (* *p* < 0.05), each group compared with model control group (^Δ^ *p* < 0.05).

It could been seen that obvious erythema, edema, oozing, and auricle swelling appeared on the right ear of mice after stimulating the Delayed Type Hypersensitivity (DTH) reaction. Mouse auricle swelling was obviously improved after treatment with SZK oil obtained by the dry distillation, baking, and ethanol extraction methods and in the paeonol control, but the mice degree of auricle swelling of the reduced pressure distillation method group grew worse. This confirmed that except for the reduced pressure distillation method, the SZK oils prepared from egg yolk oil extracted by different methods had a good effect on the degree of mice auricle swelling, which had significant differences compared with the model group (Table 3).

**Table 3:** Effects of SZK oil on auricle swelling induced by DNCB ($\bar{X}\pm \mathrm{SD}$).

Groups	Swelling Degree (g)	Swelling Rate (%)
Blank control group	0.0023 ± 0.0003	0.268 ± 0.173
Model control group	0.0115 ± 0.0060 *	0.973 ± 0.527
Dry distillation group	0.0030 ± 0.0020 ^Δ^	0.334 ± 0.132
Baking method	0.0040 ± 0.0030 ^Δ^	0.389 ± 0.287
Reduced pressure distillation group	0.0145 ± 0.0059 *	1.286 ± 0.508
Ether extraction group	0.0063 ± 0.0044 *	0.755 ± 0.393
Ethanol extraction group	0.0045 ± 0.0046 ^Δ^	0.501 ± 0.387
Petroleum ether extraction group	0.0171 ± 0.0070 *	1.208 ± 0.641
Chloroform extraction method	0.0172 ± 0.004 *	1.265 ± 0.352
Paeonol control group	0.0042 ± 0.0030 ^Δ^	0.391 ± 0.289

Note: Swelling degree = Right ear piece weight—Left ear piece weight, Swelling rate = Weight difference of left and right ear tissue/Left ear, Each group compared with blank control group (* *p* < 0.05), Each group compared with model control group (^Δ^ *p* < 0.05).

The influence of eczema on the thymus index of each group (Table 4) was: reduced pressure distillation group > chloroform extraction group > ethanol extraction group > paeonol control group > ether extraction method group > baking method group. The spleen index was not significantly different among groups.

**Table 4:** The influence of egg yolk oil on the mice immune organ weight ($\bar{X}\pm \mathrm{SD}$).

Groups	Thymus Index (%)	Spleen Index (%)
Blank control group	0.1690 ± 0.0583	0.372 ± 0.106
Model control group	0.1880 ± 0.0489	0.348 ± 0.103
Dry distillation group	0.1548 ± 0.0380	0.395 ± 0.199
Baking method	0.1276 ± 0.0756 ^Δ^	0.337 ± 0.049
Reduced pressure distillation group	0.0642 ± 0.0417 *^,Δ^	0.338 ± 0.062
Ether extraction group	0.1207 ± 0.0383 ^Δ^	0.359 ± 0.109
Ethanol extraction group	0.1043 ± 0.0479 *^,Δ^	0.329 ± 0.066
Petroleum ether extraction group	0.1420 ± 0.0394	0.323 ± 0.122
Chloroform extraction group	0.0993 ± 0.0693 *^,Δ^	0.339 ± 0.067
Paeonol control group	0.1197 ± 0.0391 ^Δ^	0.331 ± 0.068

Note: Thymus index = Thymic weight/Mice weight, Spleen index = Spleen weight/Mice weight, Each group compared with blank control group (* *p* < 0.05). Each group compared with model control group (^Δ^ *p* < 0.05).

### 2.3. Auricle Tissue Pathological Section

**Figure 2:**
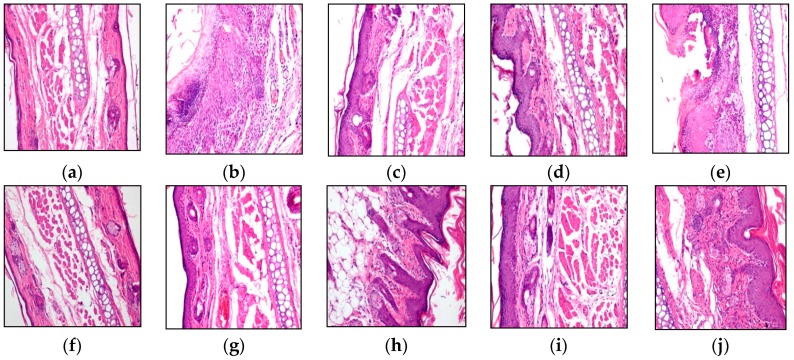
Representative histological images of the auricle tissue. (**a**) The histological image of blank control groups showing a normal epithelial tissue (magnification = 200×); (**b**) in the model control group, epithelium presented obvious proliferation and purulent secretions, infiltration of inflammation cells and telangiectasias in the corium layer, nucleus condensation, degeneration, necrosis. (magnification = 200×); (**c**) the histological image of dry distillation method group showing epithelial tissue restored to its normal form (magnification = 200×); (**d**) the histological image of the baking method group showing epithelial tissue with restored normal shape (magnification = 200×); (**e**) the histological image of the reduced pressure distillation method group showing that there were epithelial cytoplasmic vacuoles, infiltration of inflammation cells and telangiectasias in the corium layer, and nucleus necrosis (magnification = 200×); (**f**) the histological image of the ether extraction method group showing epithelial tissue restored to its normal form (magnification = 200×); (**g**) the histological image of the ethanol extraction method group showing epithelial tissue restored to its normal form (magnification = 200×); (**i**) the histological image of the chloroform extraction method group showing mild infiltration of inflammation cells (magnification = 200×); (**j**) the histological image of the paeonol control group showing epithelial tissue restored to its normal form (magnification = 200×).

The histological images of auricle tissue confirmed that the mice model of eczema was successfully established (Figure 2b). The ethanol extraction group (Figure 2g), ether extraction group (Figure 2f), paeonol control group (Figure 2i), and baking method group (Figure 2d) showed reduced edema and oozing, alleviated keratinization, lightened denaturalization and putrescence of epithelial keratinocytes as well as decreased infiltration of inflammation cells.

## 3. Discussion

The use of egg yolk oil for topical treatment (to heal fire sores) appeared in the “set proved recipe” of the Northern Zhou Dynasty for the first time, and there are documents about treating sores, fistulas, dysentery and other diseases by oral treatment with egg yolk oil. This has been documented in ancient monographs, such as “Rihuazi Bencao”, “Compendium of Materia Medica”, “Chinese Eaglewood” and “Bencao Pinhui Jingyao” [14,22,23,24]. The document “Chinese herbal medicine” summarizes that egg yolk oil mainly treats burns and scalds, otitis media, chapped skin, trauma and parasite sores, and so on [25]. Modern pharmacological studies have shown that egg yolk oil possesses analgesic, wound healing promoting, anti-oxidation, anti-aging, improving memory, lowering blood pressure and other effects [26].

This study evaluated the extraction methods of egg yolk oil according to the effects and characteristics of the egg yolk oil in eczema rehabilitation prescription (Table 5). At present, most approaches aimed at extracting egg yolk oil are based on oil yield rate and chemical ingredients content. However, Chinese medicine uses medicines made of more than one ingredient, and there is no direct linear relationship between the efficacy and one of chemical ingredient or oil yield rate index. In this study, egg yolk oil obtained by different extraction methods was used to prepare SZK oil. Its treatment efficacy was evaluated pharmacodynamically by using the mice model with acute eczema, combined with oil yield rate, characteristics, color and other indicators which provided safe and effective evidences for clinical application.

**Table 5:** The evaluation of egg yolk oil.

Preparation Methods	Evaluation Item						
	Oil Yield Rate	Efficacy for Skin Damage	Efficacy for Swell	Efficacy for Thymus	Economy and Safety Level	Color	Smell
Dry distillation method	++	+++	+++	+	++	Black Brown	Disgusting smell
Baking method	+++	++	+++	++	++	Black Brown	Disgusting smell
Reduced pressure distillation	+	−	−−	+++	+	Brown	Disgusting smell
Ether extraction method	++	+++	+	++	−	Orange	Slight egg fragrance
Ethanol extraction method	+++	+++	+++	+++	+++	Orange	Strong egg fragrance
Petroleum ether extraction method	+++	+++	−	+	++	Orange	Slight egg
Chloroform extraction method	+++	+++	−	+++	−	Orange	Slight egg

Note: the + represented a good performance and the – represented a bad one.

The oil yield rates by the chloroform extraction method, petroleum ether extraction method, baking method, and the ethanol extraction method were all above 40%. However, oil extracted by baking method is black brown and has a burnt smell, so it is not suitable for the preparation of eczema liniments. The ethanol extraction and petroleum ether extraction method product is orange in color. In particular, the ethanol extraction method resulted in a rich egg fragrance and therefore is expected to be more easily accepted by patients. Zhang [22] prepared egg yolk oil by the ethanol extraction method, and obtained a good product in terms of odor, character and pharmacological activity.

As for the pharmacodynamics, the oils extracted by the ether, ethanol and petroleum ether extraction methods showed good curative effect on eczema skin lesions in mice. The carbonization method, baking method and ethanol extraction method products showed a good effect on the auricle swelling of the mice eczema model, which was significantly different compared with the model groups. The ether, chloroform and ethanol extraction method oils can reduce the mice thymus index that is the comparable to that of the control drug paeonol. The pathological section of auricle tissue in the mice model of eczema also confirmed that curative effect and the repair effect on the epithelial defects. The pathology images of auricle tissue showed that except for the reduced pressure distillation method oil, the SZK oils prepared from egg yolk oil extracted by different methods could reduce edema and oozing, alleviate keratinization, lighten denaturalization and putrescence of epithelial keratinocytes and decrease infiltration of inflammation cells.

For economics and safety, chloroform and ethyl ether are chemically dangerous reagents, so both of them should be carefully considered if used for production, and petroleum ether is expensive, while ethanol, compared with ethyl ether, chloroform and petroleum ether, is both economic and safe. Jia [27] studied the application of ethanol in the extraction of egg yolk oil, and found that the oil yield reached 60%. The egg yolk oil was of high quality and the extraction cost was 86007 RMB/t (oil). Thus the ethanol extraction method is suitable for industrial production. 

Overall, the ethanol extraction method showed high oil yield rate, orange color, rich egg fragrance and good therapeutic effect on acute eczema. The ethanol extraction method is thus the optimal egg oil extraction method to prepare SZK thanks to its relative safety and economic feasibility. In further experiments, in order to achieve a better application to clinical studies, the unique ingredients and main components in egg yolk oil extracted by the ethanol extraction method should be studied, and the number and variety of experimental animals should be increased.

## 4. Experimental Section

### 4.1. Animals and Diet

All procedures in this study were approved by the Hunan University of Chinese Medicine Animal Care and Use Committee and were in compliance with the Guide for the Care and Use of Laboratory Animals. One hundred Kuming mice (50 males and 50 females, four weeks of age, *n* = 10 for each group) were obtained from East innovation experimental animal science and Technology Services Department (Changsha, China). Animal qualified number was SCXK (Xiang) 2009-0013. They were allowed to acclimate for one week. All mice were maintained at 21 °C on a 12-h light/12-h dark cycle with free access to water and food.

### 4.2. Experimental Reagents

Ether was purchased from Tianjin Kemiou Chemical Reagent Co. Ltd. (Tianjin, China). Petroleum ether was obtained from Tianjin Fu Yu Fine Chemical Co. Ltd. (Tianjin, China). Anhydrous ethanol was purchased from Tianjin HengXing Chemical Reagent Co. Ltd. (Tianjin, China). Chloroform was purchased from Kaixin Chemical Reagent Co. Ltd. (Hengyang, China). 2,4-Dinitrochlorobenzene was purchased from Tianjin Guangfu Fine Chemical Research Institute (Tianjin, China). Acetone was obtained from Shenyang Yuanhang Chemical Co. Ltd. (Shenyang, China). Kuh-seng exctracts and borneol were purchased from Cunrentang Co. Ltd. (Zhenjiang, China). Paeonol was obtained from Ningbo Tianzhen Pharmaceutical Co. Ltd. (Ningbo, China). 

### 4.3. Primary Instruments

Water-bath pot (HH-S), Circulating water vacuum pump (SHZ-DIII), Rotary evaporator (RE-2000) and constant temperature heating magnetic stirrer (III) were purchased from Gongyi City Yuhua Instrument Co. Ltd. (Gongyi, China). Far infrared fast constant temperature drying box was obtained from Shanghai Yuejin Medical Optical Instruments Factory (Shanghai, China). TDl0001 electronic balance was purchased from YuYao Jinnuo Balance Instrument Co. Ltd. (Yuyao, China). TG328B analytical balance was purchased from Shanghai Banlance Instrument Technical Co. Ltd. (Shanghai, China). Animal shaver was obtained from Ningbo Daxie Development Zone Heng Qin Electric Appliance Factory (Ningbo, China). Olympus electron microscope was purchased from Olympus Corporation (Tokyo, Japan).

### 4.4. Extraction of Egg Yolk Oil

Fresh eggs were bought from a local Chinese market, and then they were cooked by bringing tap water to a boil and then kept simmering for 20 min. After being cooled in tap water for 15 min, eggs were shelled and egg yolk and egg white were separated. Wet pellets from egg were produced, through 14 mesh sieves, and then placed into the constant temperature drying box at 37 °C for 12–20 h until the yolk granules dried. The experimental samples were kept in sealed plastic bags in refrigerator at a temperature of +4 °C until experiment. The egg yolk oil was extracted by different methods as follows:

1. Dry distillation method: egg yolk powder (174 g) was gently heated in the crucible until the water evaporated, and then heated with high heat to 275 °C until the egg yolk oil precipitated out. The oil obtained was separated from the residue with an asepsis gauze and weighed with an electronic balance.
2. Baking method: egg yolk powder (100 g) was put onto a Buchner funnel lined with 60 mesh barbed wire and covered with a dish. The bottom of the funnel was put into a beaker, and placed in a constant temperature drying box, which was heated slowly up to 240 °C for 30 min. The obtained egg yolk oil was collected and weighed in the beaker.
3. Reduced pressure distillation method: egg yolk (100 g) was put into four round bottom flasks and distilled on the electric heating set, which was adjusted to 250 °C. The egg yolk was distilled under reduced pressure (0.05–0.06 MPa) for 4 h in. The oil was collected and weighed.
4. Ether extraction method: egg yolk powder (70 g) was added to ether (560 mL) and the oil was extracted under water bath heating at room temperature for 1.5 h. The remaining egg yolk powder was extracted again by ether (420 mL) for 1 h. After the removal of ether, the oil was collected and weighed.
5. Ethanol extraction method: egg yolk powder (100 g) was added to 95% ethanol (800 mL) and extracted under water bath heating at 85 °C for 1.5 h, followed by addition of 95% ethanol (600 mL) to the remaining powder for 1 h. After the removal of ethanol, the oil was collected and weighed.
6. Petroleum ether extraction method: egg yolk powder (100 g) was added to petroleum ether (800 mL) and extracted under water bath heating at 65 °C for 1.5 h, and then more petroleum ether (600 mL) was added to the remaining powder for extraction for 1 h more. After removal of petroleum ether, the oil was collected and weighed.
7. Chloroform extraction method: egg yolk powder (100 g) was placed in a Soxhlet extractor filled with chloroform (200 mL) and heated in a 65 °C water bath for 1 h. The extraction was repeated four times until the egg yolk was white. After the removal of chloroform, the oil was collected and weighed.

All these extraction experiments were repeated five times according to the above extraction methodx. Oil yield rate % = (mass of egg yolk oil/mass of egg yolk powder) × 100%.

### 4.5. Preparation of SZK Oil

SZK oil was obtained by mixing kuh-seng extracts (1.5 g), and borneol (0.5 g) with egg yolk oil (20 g) extracted according to the different extraction methods.

### 4.6. Mice Model of Eczema

1. Grouping and experimental preparation: after one week of adaptive feeding, the 100 mice were randomly divided into 10 groups, including blank control group, model control group, dry distillation method group, baking method group, reduced pressure distillation method group, ether extraction group, ethanol extraction group, petroleum ether extraction group, chloroform extraction group and paeonol (as positive drug) control drug group. Each group included five male mice and five female mice. All mice were sheared with an area of 3 cm × 3 cm on the abdomen and 1 cm × 2 cm on the left back.
2. Sensitization: Except for the blank control group, 50 μL 5% DNCB acetone solution was evenly smeared onto the mice abdomen sheared zone to sensitize on the 1st day, and repeated on the 2nd day.
3. DTH reaction stimulation: 10 μL and 15 μL of 1% DNCB were evenly smeared on the right ear in mice (both sides) and left back sheared zone on the 7th day, respectively. The ear and back skin of the blank control group were smeared similarly with DNCB All mice were stimulated again with a double dose on the 9th and 10th day.

### 4.7. Oil Administration

After 24 h of stimulation, each group of mice was treated with the corresponding drugs on the right ear and back sheared zone. The inside of the right ear and back sheared zone were treated with 0.05 mL and 0.1 mL SZK oil, respectively, twice per day for 5 days. The mice were killed 2 h after the last treatment.

### 4.8. Detection Index

#### 4.8.1. Visual Observation

The skin changes, such as erythema and edema, on the back of experimental mice were observed daily since the start of drug treatment. The mice were scored two times per week according to the severity of erythema (No erythema as 0, slight erythema, edema and oozing or keratinization as 1, obvious erythema, edema and oozing as 2, moderately severe erythema and edema, obvious oozing as 3, severe erythema and edema, serious oozing, obvious keratinization and escharosis as 4).

#### 4.8.2. Auricle Swelling Degree

The auricular tissue samples were immediately removed using a 7 mm hole puncher when the mice were executed and then weighed. The weight difference between the left and right ear was defined as swelling degree, and the ratio (%) of the weight difference of the left ear and right ear was defined as swelling rate:

Swelling degree = Right ear piece weight − Left ear piece weight,

Swelling rate = Weight difference of left and right ear tissue/Left ear.

#### 4.8.3. Thymus Index and Spleen Index

The thymus and spleen of each group of mice were weighed after the mice were executed and the thymus index and spleen index were calculated, respectively:

Thymus index = Thymic weight/Mice weight,

Spleen index = Spleen weight/Mice weight.

#### 4.8.4. Pathological Section of Auricle Tissue

The auricle tissues from the right ear of mice were cut into slices, dyed by hematoxylin and eosin (HE) staining, and observed under microscope.

### 4.9. Statistical Analysis

Results are expressed as means ± standard deviation of the mean ($\bar{X}\pm \mathrm{SD}$) for five independent experiments. A two-tailed Student’s *t* test was used for statistical comparisons between any two specific experimental groups. Multiple group comparisons were done by ANOVA using a least significant difference *post hoc* analysis (SPSS 16.0 SPSS Inc., Chicago, IL, USA). *p* < 0.05 was considered statistically significant.

## 5. Conclusions

Using the egg yolk powder as raw material, egg yolk oil was prepared by the distillation, baking, reduced pressure distillation, ether extraction, ethanol extraction, petroleum ether extraction and chloroform extraction methods, respectively. SZK oil was then made by using the obtained egg yolk oils to evaluate the egg yolk oil extraction methods by their pharmacodynamic indexes combined with oil yield rate, characteristics, color, smell, *etc.* The results indicated that ethanol extraction was the optimal extraction method. This method preserves the main functions of the eczema rehabilitation prescription, is suitable for preparation production and has a good pharmacodynamic performance.
